# Community‐led monitoring: a voice for key populations in Zimbabwe

**DOI:** 10.1002/jia2.25925

**Published:** 2022-07-12

**Authors:** Tatenda Makoni, Gilton Kadziyanhike, Clarence Mademutsa, Martha Mlambo, Kalonde Malama

**Affiliations:** ^1^ Zimbabwe National Network of People Living with HIV (ZNNP+) Harare Zimbabwe; ^2^ University of Toronto Factor‐Inwentash Faculty of Social Work Toronto Ontario Canada

**Keywords:** PLHIV, Key populations, ZNNP+ CTO model, quality improvement, Men who have sex with men, sex workers

1

Community‐led monitoring has the potential to be an effective mechanism for organized and systematic advocacy that is well appreciated by service providers. Engaging peers in service delivery is an effective strategy to reduce stigma among key populations. In this Viewpoint, we describe an example from Zimbabwe where a community treatment observatory model has been implemented to systematically provide services and track results for people living with HIV and key populations. It is a peer‐based model that has been adopted by the Zimbabwe National Network of People living with HIV (ZNNP+).

With an HIV prevalence of 12.8%, which translates to 1.4 million people living with HIV, Zimbabwe remains one of the most HIV endemic countries in sub‐Saharan Africa [1]. Recent data show that key populations, who include sex workers, men who have sex with men, transgender people, incarcerated people and various other groups, account for 62% of the new HIV infections [1]. Structural factors, such as pervasive stigma, discrimination, human rights violations, physical, emotional and sexual violence, are some of the challenges key populations face [2].

In Zimbabwe, the legal context marginalizes key populations by criminalizing same‐sex relationships. The Zimbabwean penal code outlaws same‐sex relations under the “sodomy” and “indecent act” clauses, and Section 81 prohibits the solicitation of sex work [3]. Further, the social environment in Zimbabwe engenders attitudes that impede key populations’ access to healthcare; for instance, key populations in Zimbabwe believe that healthcare would be more accessible if they conformed to “sexual norms,” and that the stigmatizing attitudes of healthcare workers towards key populations affect the quality of care offered [4]. Key populations, therefore, carry a disproportionate burden of HIV, tuberculosis, sexually transmitted infections, poor mental health and other health concerns [5].

Community‐led monitoring has been an effective instrument for providing HIV prevention and care to key populations while ensuring services are stigma‐free for all recipients. An example of a community‐led monitoring system is the community treatment observatory. This observatory employs community members, such as people living with HIV or those representing key populations without HIV to collect data from their peers about the quantity and quality of HIV prevention, care and treatment services within communities [6]. The collected data are then analysed and used to inform community‐driven healthcare services [6]. Such models have had success in Sierra Leone, where, within a year of implementing a community‐led monitoring system, HIV testing increased by 85% among men who have sex with men, 100% among female sex workers, 96% among people who inject drugs and 90% among young people; while antiretroviral therapy uptake increased by 93% among people living with HIV [7].

Stigma and discrimination negatively impact the quality of life of people living with HIV in various ways, including detracting from mental health and inhibiting access to care [8, 9, 10]. ZNNP+ is a national umbrella body that supports the efforts to reduce stigma and discrimination against people living with HIV. To destigmatize care and improve health outcomes for people living with HIV, ZNNP+ utilizes a community treatment observatory model. The model is based on the principle of including often marginalized populations in the decision‐making process around their healthcare. In doing so, healthcare systems can better ensure the rights of key populations and provide stigma‐free care for all.

The community treatment observatory model at ZNNP+ involves recruiting and training self‐disclosed community cadres living with HIV to collect data from their peers, coordinate community dialogues, and engage with community leaders and health facilities. The cadres include mentor mothers, community HIV and AIDS support agents and key population peer supporters who are sex workers and men who have sex with men. Mentor mothers are women living with HIV who have successfully delivered infants who are HIV negative through prevention of mother‐to‐child transmission and help their peers achieve the same. Community HIV and AIDS support agents are self‐disclosed people living with HIV who help their peers access services that improve their quality of life. The community treatment observatory model aligns with recent calls to shift away from viral suppression‐oriented care models towards more person‐centred approaches that focus on ensuring the health of people living with HIV throughout their lives [11].

The community treatment observatory model plays two roles: ensuring data‐driven evidence on issues affecting key populations and providing key populations with a platform to share their views confidentially. Community cadres are trained to do community‐led monitoring using mobile data collection. Data collection consists of in‐person qualitative and quantitative interviews using the customer satisfaction survey questionnaires that cover availability, affordability, accessibility, acceptability and appropriateness issues. This provides valuable insights into how key populations perceive the way they are being served at facilities. Community cadres collect data on satisfaction with time spent at health facilities, stigma and discrimination at facilities and in the community, privacy during consultations, and frequency and duration of antiretroviral treatment stock‐outs.

In our experience, community‐led monitoring has proved to be an effective mechanism for organized and systematic advocacy for the health and rights of key populations. Engaging peers in service delivery is an effective strategy to reduce stigma among key populations. Getting feedback from receivers of care could encourage healthcare workers to improve person‐centred service and address issues, such as stigma, that cause low uptake of services and poor health outcomes. The example of ZNNP+ illustrates that, when properly resourced, communities can deliver crucial, destigmatized services to their peers. These services ultimately aim to ensure that health systems, governments and other stakeholders are accountable for meeting the needs and upholding the rights of key populations.

## COMPETING INTERESTS

All authors except KM are employees of ZNNP+.

## AUTHORS’ CONTRIBUTIONS

TM, CM and MM contributed to the conceptualization and writeup of the manuscript. GK contributed to the conceptualization, writeup and revision of the manuscript. KM contributed to the writeup and revision of the manuscript. TM, GK, CM, MM and KM all read and approved the final manuscript.

## FUNDING

There was no specific funding for this Viewpoint.
